# Association between corpus callosum microstructure and upper limb motor outcome in moderate-to-severe ischemic stroke patients at 3–6 months post-stroke

**DOI:** 10.3389/fneur.2026.1852619

**Published:** 2026-08-18

**Authors:** Jaeun Koo, Ah Yeon Lee, Eunjoo Lee, So-Youn Chang, Youngkook Kim

**Affiliations:** 1 Department of Rehabilitation Medicine, Yeouido St. Mary’s Hospital, College of Medicine, The Catholic University of Korea, Seoul, Republic of Korea; 2 Department of Rehabilitation Medicine, Seoul St. Mary’s Hospital, College of Medicine, The Catholic University of Korea, Seoul, Republic of Korea

**Keywords:** biomarkers, corpus callosum, diffusion tensor imaging, motor function, stroke, upper extremity

## Abstract

**Background:**

Corticospinal tract (CST) integrity is a well-established imaging correlate of post-stroke motor outcome but does not fully explain interindividual variability, particularly in moderate-to-severe impairment. The corpus callosum (CC) may provide complementary structural information. This study examined whether early subacute callosal microstructure is associated with upper limb motor outcome and recovery.

**Methods:**

We retrospectively analyzed 38 patients with moderate-to-severe middle cerebral artery stroke who underwent diffusion tensor imaging during the early subacute phase. Fractional anisotropy (FA) values of the CST and CC were obtained. Upper limb motor function was assessed using the Shoulder Abduction and Finger Extension (SAFE) score. Simple linear regression and hierarchical multiple linear regression analyses examined associations of corrected CC FA (CC-FA ~ corrected~) with SAFE scores and ΔSAFE (change in SAFE score from baseline to 6 months).

**Results:**

CST FA laterality index (CST FALI), lesion volume, and CC-FA ~ corrected~ were significantly associated with SAFE scores at 3 and 6 months post-stroke. In hierarchical multiple regression models, CC-FA ~ corrected~ of the genu showed a consistent nominal incremental contribution beyond CST FALI, age, and lesion volume at both timepoints (3 months: ΔR^2^ = 0.061, adjusted R^2^ = 0.600; 6 months: ΔR^2^ = 0.065, adjusted R^2^ = 0.646), though these findings require confirmation in larger prospective cohorts. The body and splenium did not reach statistical significance after accounting for these covariates. In exploratory analysis, CC-FA ~ corrected~ of the genu was the only variable significantly associated with ΔSAFE (R = 0.396, *p* = 0.041); CST FALI was not (*p* = 0.097).

**Conclusion:**

CC-FA ~ corrected~ of the genu showed a consistent nominal association with upper limb motor outcome beyond CST integrity in moderate-to-severe stroke, suggesting a complementary role of callosal microstructure alongside CST integrity, with an exploratory association also observed for the magnitude of motor improvement over time. Early combined assessment of CC and CST integrity during the subacute phase may provide preliminary information regarding post-stroke motor outcomes.

## Introduction

Clinical measures have limited ability to estimate upper limb motor outcome in patients with severe post-stroke impairment, where conventional motor scores often exhibit floor effects (1). Neuroimaging biomarkers have therefore been proposed as objective markers associated with motor outcomes in this population. Among these, the structural integrity of the corticospinal tract (CST) is a well-established imaging correlate of upper limb motor outcome across varying levels of impairment (2–4). However, CST integrity alone does not fully account for the variability in motor performance among patients with severe motor deficits, highlighting the need to consider additional structural pathways (5).

The corpus callosum (CC), the principal white matter tract connecting the two hemispheres, may serve as a structural substrate supporting interhemispheric communication relevant to motor performance, consistent with prior neurophysiological studies implicating interhemispheric processes in post-stroke motor recovery (6, 7). Previous studies have reported that greater CC integrity is associated with better post-stroke motor outcomes (8–11), with particularly strong associations observed in patients with severe deficits (5, 12). However, most of these studies were conducted in chronic stroke populations or examined cross-sectional relationships. Whether early subacute CC microstructure contributes additional explanatory information beyond CST integrity for later upper limb motor outcome remains unclear. This is a clinically relevant gap, as the early subacute phase coincides with the initiation of inpatient rehabilitation—a time point at which structural biomarkers may provide preliminary information to support individualized treatment planning and goal-setting, pending prospective validation.

The present study aimed to examine whether the structural integrity of the CC measured during the early subacute phase is independently associated with upper limb motor outcome at 3 and 6 months post-stroke in patients with moderate-to-severe stroke. We also examined whether CC microstructure was associated with the magnitude of motor improvement over time. We hypothesized that higher CC integrity would be positively associated with both better upper limb motor performance and greater motor recovery in this population.

## Materials and methods

### Study cohort

This retrospective cohort study included patients admitted to the inpatient rehabilitation facility (IRF) of our institution after acute stroke management. A total of 38 individuals with middle cerebral artery (MCA) territory stroke who underwent brain diffusion tensor imaging (DTI) during their rehabilitation admission between January 2019 and February 2022 were analyzed. The patients who met the following criteria were included: (1) age above 18 years; (2) first-ever ischemic stroke restricted to the MCA territory; (3) moderate-to-severe disabilities, defined as a modified Rankin scale ≥3, and (4) early subacute phase of stroke recovery, defined as 7 days to 3 months after stroke onset (13). The exclusion criteria were as follows: (1) a bi-hemispheric stroke, (2) a history of prior ischemic or hemorrhagic stroke, or (3) history of additional neurological disorders beyond stroke.

### Ethics statement

The institutional review board of our institution approved the protocols for this study, which were in accordance with the Declaration of Helsinki. The requirement for informed consent was waived because this study was retrospective, involved existing data, and there were no concerns regarding subject confidentiality.

### Functional assessments

The hemiplegic upper limb motor function was evaluated using the Shoulder Abduction and Finger Extension (SAFE) score at baseline, 3 months and 6 months after stroke onset: at baseline (approximately 7–14 days post-stroke, coinciding with IRF admission), at 3 months, and at 6 months after stroke onset. This measure reflects voluntary muscle strength by summing the Medical Research Council (MRC) grades for shoulder abduction and finger extension, each scored from 0 (no visible contraction) to 5 (normal strength) (14). The total SAFE score therefore ranges from 0 to 10, with higher values indicating better upper limb motor performance. It is a simple and reliable measure, which is incorporated into the Predict Recovery Potential (PREP)1 and PREP2 algorithms for post-stroke motor outcome (3, 15).

### DTI acquisition

DTI was performed using a 3.0-T magnetic resonance imager (MAGNETOM® Skyra; Siemens, Erlangen, Germany) with a six-channel head coil. Single-shot spin-echo echo-planar images were acquired across 70 interleaved axial slices (thickness, 2.0 mm; no gap; TR/TE = 14,300/84 ms; FOV = 256 × 256 mm^2^; matrix = 128 × 128; voxel size = 2 × 2 × 2 mm^3^; NEX = 1). Diffusion-sensitizing gradients were applied in 30 noncollinear directions (b = 1,000 s/mm^2^) with one b = 0 reference image.

### Image processing

The images were processed using the FMRIB Software Library (version 5.0.9).^1^ The source data were corrected for eddy currents and head motion by registration to the first b = 0 image using affine transformation. Additionally, pixel-wise outlier detection was applied to compensate for residual artifacts including eddy current distortion, head motion, and cardiac pulsation artifacts. Fractional anisotropy (FA) maps were prepared using DTIFIT. All FA images were normalized to 1 mm^3^ MNI152 standard space using nonlinear registration prior to atlas-based extraction.

### DTI analysis

**Figure 1:**
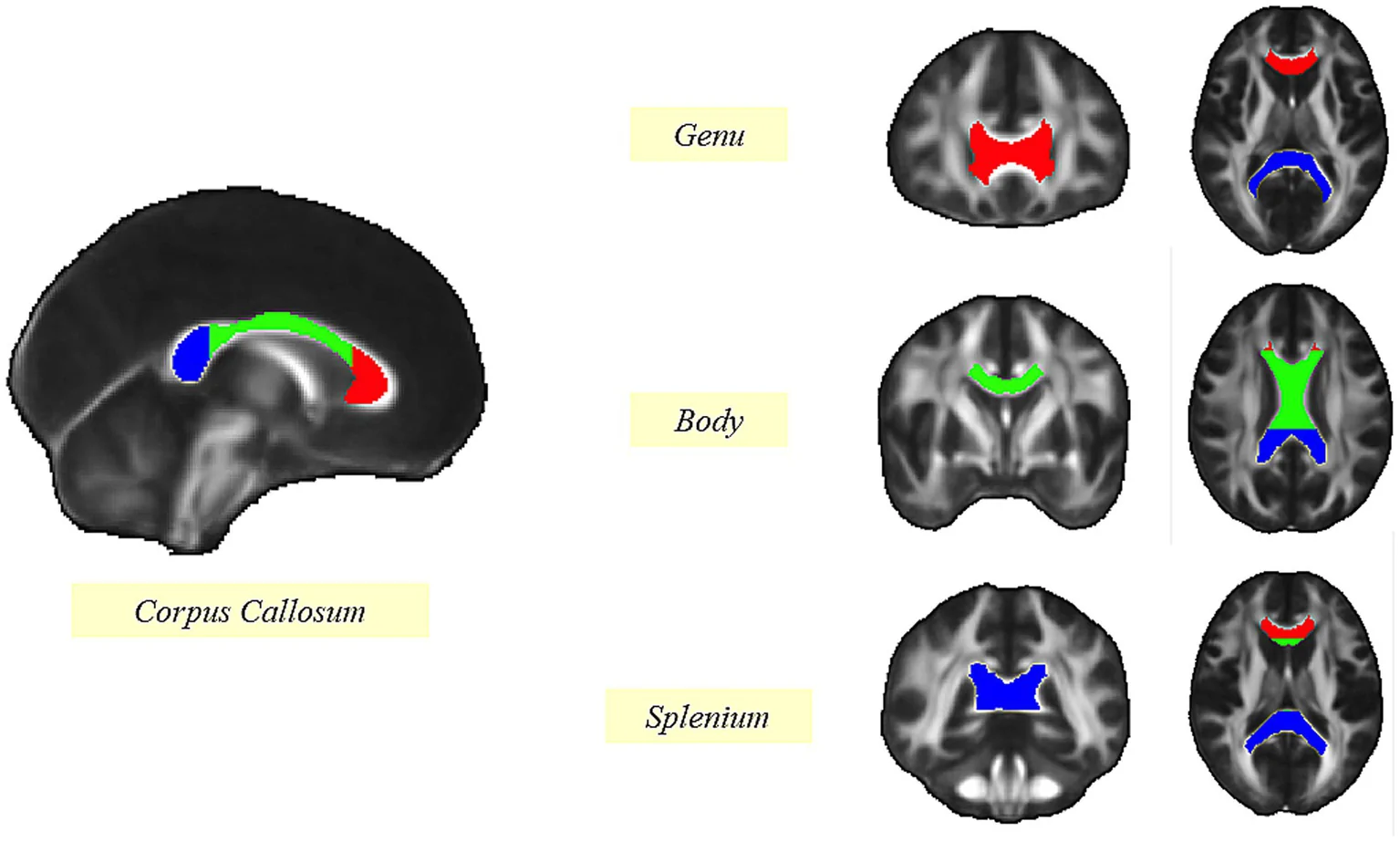
Regions of interest (ROIs) used to evaluate the microstructural integrity of the genu, body, splenium of the corpus callosum (CC) on diffusion tensor imaging.

FA values of the CST were obtained using a region-of-interest (ROI) approach at the pontomedullary junction on axial slices of the color FA maps (4) (Figure 1). ROIs were manually drawn on individual diffusion space images by a single experienced physician who was blinded to all clinical outcome data at the time of measurement, consistent with the blinding practice applied in prior publications from our research group (16, 17). As all measurements were performed by a single rater applying a consistent, predefined protocol established across multiple prior studies from our group (18), formal inter-rater reliability testing was not conducted in the present study. However, in a prior study from our group using the identical DTI protocol and ROI approach at the same institution, inter-rater reliability between two experienced raters was ICC = 0.855 (95% CI: 0.733–0.919) (18), supporting the reliability of the measurement approach applied here. The structural integrity of the CST was expressed as the FA laterality index (FALI), calculated by the following formula: (FA_damaged_ − FA_non-damaged_) / (FA_damaged_ + FA_non-damaged_). A lower FALI represents greater asymmetry and lesser structural reserve on the damaged side.

For the CC, tract-of-interest analysis was performed using the Johns Hopkins University DTI White Matter Atlas, including the genu, body, and splenium of the CC (19). FA values were extracted following atlas-based tract-of-interest analysis applied to FA images registered to 1 mm^3^ MNI152 standard space using nonlinear registration, consistent with the approach used in prior work from our group (20, 21). As the CC is one of the tracts that are more vulnerable to age-related microstructural decline, FA values were normalized to those of the non-lesioned CST to derive a corrected CC FA (CC-FA ~ corrected~) according to the following formula: (CC FA) / (non-damaged CST FA). The non-damaged CST served as a reference tract due to its relative preservation and established measurement reliability (4). Lesion volumes were manually delineated and measured.

### Statistical analysis

Normality of all variables was assessed using the Kolmogorov–Smirnov and Shapiro–Wilk tests. To address the multiplicity of analyses across CC subregions and follow-up timepoints, analyses were prespecified as primary, secondary, or exploratory prior to data interpretation. The strength of the association between CC-FA ~ corrected~ and SAFE scores at 3 and 6 months post-stroke was assessed using simple linear regression analyses as secondary descriptive analyses. The primary analyses comprised hierarchical multiple linear regression models examining whether CC-FA ~ corrected~ of each CC subregion provided incremental explanatory value for upper limb motor outcome beyond established covariates at each timepoint. In the first step, age, lesion volume, and CST FALI were entered simultaneously as forced covariates. In the second step, CC-FA ~ corrected~ of each CC subregion (genu, body, and splenium) was entered separately in three independent models. The incremental contribution of each subregion was evaluated using the change in R^2^ (ΔR^2^), standardized *β* coefficients, 95% confidence intervals, and *p* values. As six hierarchical models were constructed across three subregions and two timepoints, findings from the primary analyses should be interpreted in the context of multiple comparisons; a consistent pattern of significance across timepoints, rather than isolated p values, was considered the primary criterion for interpreting subregion-specific contributions. In an exploratory analysis, ΔSAFE (change in SAFE score from baseline, assessed at approximately 7–14 days post-stroke, to 6 months post-stroke) was examined against clinical and neuroimaging variables using simple linear regression. Results of the exploratory analysis are considered hypothesis-generating and require confirmation in future studies. Multicollinearity among predictors was assessed using the variance inflation factor. All analyses were two-tailed, and a p value < 0.05 was considered statistically significant. Statistical analyses were performed using SPSS (version 29.0.2; IBM Corp., Armonk, NY, USA).

## Results

### Demographic and clinical characteristics

The demographic and clinical characteristics of the participants are presented in Table 1. The mean National Institutes of Health Stroke Scale (NIHSS) score at stroke onset was 11.00 ± 5.41, indicating moderate-to-severe stroke. The mean modified Barthel index score was 25.03 ± 24.40, indicating that most patients required moderate assistance or more. Mean DTI acquisition interval was 27.08 ± 20.55 days post-stroke.

**Table 1:** Demographic and clinical characteristics of the patients.

Characteristics	Patients (*N* = 38)
Age, years	73.74 ± 11.93
Female sex, n (%)	19 (50)
Stroke characteristics	
Left hemisphere lesion, n (%)	24 (63.16)
Lesion volume, ${\mathrm{cm}}^{3}$	56.74 ± 65.46
Clinical characteristics	
Fazekas scale, total^*^ (range 0–6)	3 (1–4)
NIHSS score, initial	11.00 ± 5.41
SAFE score, initial^*^	2 (0–5)
MMSE (range 0–30) at baseline	14.68 ± 9.88
MBI (range 0–100) at baseline	25.03 ± 24.40
Modified Rankin Scale^*^ at baseline	5 (4–5)
Data acquisition	
DTI acquisition, days^†^	27.08 ± 20.55

NIHSS, National Institutes of Health Stroke Scale; SAFE, Shoulder Abduction and Finger Extension; MMSE, Mini-Mental State Examination; MBI, Modified Barthel Index; DTI, diffusion tensor imaging. *Median value (interquartile range). ^†^Days since stroke onset. Values are expressed as the mean ± standard deviation, unless otherwise stated. Baseline assessments were obtained at approximately 7–14 days post-stroke, coinciding with IRF admission.

### Models to examine upper limb motor outcome at 3 months post-stroke

Simple linear regression analyses (secondary descriptive analyses) are presented in Table 2, and hierarchical regression models (primary analyses) are presented in Table 3. At 3 months after stroke, simple linear regression analyses revealed that the CST FALI (R = 0.666, *p* < 0.001), lesion volume (R = 0.426, *p* = 0.009), and CC-FA ~ corrected~ of the genu (R = 0.371, *p* = 0.024), body (R = 0.395, *p* = 0.016), and splenium (R = 0.392, *p* = 0.016) were each significantly associated with SAFE scores (Table 2A).

**Table 2:** Simple linear regression analyses examining associations between clinical variables, neuroimaging biomarkers, and the Shoulder Abduction and Finger Extension (SAFE) score.

	R	$R^{2}$	*t*	*p*-value
(A) 3 Months post-stroke				
Clinical variables				
Age	0.287	0.082	1.770	0.085
Lesion volume	0.426	0.182	−2.787	0.009
Neuroimaging biomarkers				
CST FALI^*^	0.666	0.443	5.275	< 0.001
Corpus callosum				
Genu^†^	0.371	0.138	2.364	0.024
Body^†^	0.395	0.156	2.545	0.016
Splenium^†^	0.392	0.154	2.522	0.016
(B) 6 Months post-stroke				
Clinical variables				
Age	0.265	0.070	1.373	0.182
Lesion volume	0.457	0.209	−2.568	0.017
Neuroimaging biomarkers				
CST FALI^*^	0.700	0.490	4.901	< 0.001
Corpus callosum				
Genu^†^	0.453	0.205	2.541	0.018
Body^†^	0.474	0.225	2.694	0.012
Splenium^†^	0.525	0.276	3.084	0.005

t, t test statistic; CST FALI, FA laterality index of the corticospinal tract (CST) measured at the pontomedullary junction; FA, fractional anisotropy. *CST FALI = (FA_damaged_ − FA_non-damaged_) / (FA_damaged_ + FA_non-damaged_). ^†^Corrected FA values using non-damaged CST FA.

**Table 3:** Multiple linear regression models examining the association between the shoulder abduction and finger extension (SAFE) score and structural integrity of the corpus callosum subregions.

Models	Adjusted *R^2^*	Part *R^2 ||^*	Significant variables	Unstandardized		Standardized		*p*-value^*^
				B (95% CI)	SE	β	*t*	
(A) 3 Months post-stroke								
Age + Lesion volume + CST FALI ^†^	0.539^‡^		CST FALI^§^	15.583 (9.141–22.024)	3.166	0.579	4.922	< 0.001
			Age	0.065 (0.008–0.122)	0.028	0.262	2.312	0.027
			Lesion volume	−0.012 (−0.023–−0.002)	0.005	−0.273	−2.321	0.027
Genu of corpus callosum^†^	0.600^‡^ (ΔR^2^ 0.061)		CST FALI^§^	15.077 (9.058–21.096)	2.955	0.561	5.102	< 0.001
			Age	0.088 (0.032–0.144)	0.028	0.357	3.176	0.003
			Lesion volume	−0.005 (−0.017–0.006)	0.006	−0.118	−0.936	0.356
		0.067	Genu	9.022 (1.562–16.482)	3.662	0.320	2.463	0.019
Body of corpus callosum^†^	0.566^‡^ (ΔR^2^ 0.027)		CST FALI^§^	14.406 (8.003–20.809)	3.143	0.536	4.583	< 0.001
			Age	0.071 (0.015–0.127)	0.027	0.288	2.595	0.014
			Lesion volume	−0.010 (−0.020–0.001)	0.005	−0.216	−1.822	0.078
		0.037	Body	6.127 (−0.989–13.243)	3.493	0.210	1.754	0.089
Splenium of corpus callosum ^†^	0.567^‡^ (ΔR^2^ 0.028)		CST FALI^§^	14.171 (7.719–20.622)	3.167	0.527	4.474	< 0.001
			Age	0.073 (0.017–0.129)	0.027	0.296	2.658	0.012
			Lesion volume	−0.010 (−0.021–0.001)	0.005	−0.225	−1.923	0.063
		0.038	Splenium	4.938 (−0.704–10.580)	2.770	0.214	1.783	0.084
(B) 6 Months post-stroke								
Age + Lesion volume + CST FALI ^†^	0.581^‡^		CST FALI^§^	17.738 (9.889–25.588)	3.794	0.610	4.675	< 0.001
			Age	0.049 (−0.019–0.116)	0.033	0.191	1.498	0.148
			Lesion volume	−0.016 (−0.028–−0.003)	0.006	−0.333	−2.567	0.017
Genu of corpus callosum^†^	0.646^‡^ (ΔR^2^ 0.065)		CST FALI^§^	16.742 (9.450–24.035)	3.516	0.576	4.761	< 0.001
			Age	0.073 (0.007–0.139)	0.032	0.287	2.302	0.031
			Lesion volume	−0.007 (−0.021–0.008)	0.007	−0.142	−0.975	0.340
		0.071	Genu	10.455 (0.946–19.965)	4.585	0.345	2.280	0.033
Body of corpus callosum^†^	0.601^‡^ (ΔR^2^ 0.02)		CST FALI^§^	16.574 (8.710–24.438)	3.792	0.570	4.371	< 0.001
			Age	0.052 (−0.014–0.118)	0.032	0.204	1.632	0.117
			Lesion volume	−0.011 (−0.025–0.003)	0.007	−0.239	−1.681	0.107
		0.032	Body	7.125 (−3.053–17.302)	4.908	0.210	1.452	0.161
Splenium of corpus callosum^†^	0.625^‡^ (ΔR^2^ 0.044)		CST FALI^§^	15.464 (7.626–23.302)	3.780	0.532	4.091	< 0.001
			Age	0.059 (−0.006–0.124)	0.031	0.231	1.885	0.073
			Lesion volume	−0.011 (−0.024–0.002)	0.006	−0.237	−1.788	0.088
		0.053	Splenium	7.270 (−0.571–15.111)	3.781	0.269	1.923	0.068

B, unstandardized beta coefficients; SE, standard error; β, standardized beta coefficients; t, t test statistic; CST FALI, FA laterality index of the corticospinal tract (CST) measured at the pontomedullary junction; FA, fractional anisotropy. *indicates the significance of independent variable. †corrected FA values using non-damaged CST FA. ‡indicates significant models with *p* < 0.001. §CST FALI = (FA_damaged_ − FA_non-damaged_) / (FA_damaged_ + FA_non-damaged_). || represents the squared semipartial correlation and indicates the unique proportion of variance in the outcome explained by each predictor after accounting for the other variables in the model.

In hierarchical multiple regression models, the genu showed a nominally significant incremental contribution beyond covariates entered in the first step (age, lesion volume, and CST FALI) at 3 months (ΔR^2^ = 0.061, *p* = 0.019), with an adjusted R^2^ of 0.600 (Table 3A). The body and splenium did not reach statistical significance in the second step (*p* = 0.089 and *p* = 0.084, respectively).

### Models to examine upper limb motor outcome at 6 months post-stroke

At 6 months post-stroke, simple linear regression analyses revealed that the CST FALI (R = 0.700, *p* < 0.001), lesion volume (R = 0.422, *p* = 0.045), and CC-FA ~ corrected~ of the genu (R = 0.453, *p* = 0.018), body (R = 0.474, *p* = 0.012), and splenium (R = 0.525, *p* = 0.005) were each significantly associated with SAFE scores (Table 2B).

In the hierarchical models, the genu again showed a nominally significant incremental contribution beyond covariates entered in the first step (ΔR^2^ = 0.065, *p* = 0.033), with an adjusted R^2^ of 0.646 (Table 3B). The body and splenium did not reach statistical significance in the second step (*p* = 0.161 and *p* = 0.068, respectively). Across both timepoints, CST FALI was the strongest predictor in the first step, and CC-FA ~ corrected~ of the genu was the only subregion to show a consistent nominal incremental contribution after accounting for CST integrity and other covariates, with numerically higher adjusted R^2^ values compared to the body and splenium models at both timepoints.

### Exploratory analysis of change in SAFE score

In an exploratory analysis examining motor improvement over time, the change in SAFE score between baseline and 6 months (ΔSAFE) was calculated and analyzed using simple linear regression analysis (Table 4; Supplementary Figure 1). Among the clinical and neuroimaging variables examined, CC-FA ~ corrected~ of the genu showed a significant association with ΔSAFE (R = 0.396, *p* = 0.041), whereas age, lesion volume, CST FALI, and CC-FA ~ corrected~ of the body and splenium were not significantly associated with ΔSAFE.

**Table 4:** Simple linear regression analysis examining associations between ΔSAFE (change in SAFE score from baseline to 6 months) and clinical and neuroimaging variables.

	R	$R^{2}$	*t*	*p*-value
Clinical variables				
Age	0.284	0.080	−1.478	0.152
Lesion volume	0.330	0.109	−1.746	0.093
Neuroimaging biomarkers				
CST FALI^*^	0.326	0.106	1.724	0.097
Corpus callosum				
Genu^†^	0.396	0.156	2.153	0.041
Body^†^	0.237	0.056	1.217	0.235
Splenium^†^	0.196	0.038	0.998	0.328

t, t test statistic; CST FALI, FA laterality index of the corticospinal tract (CST) measured at the pontomedullary junction; FA, fractional anisotropy. *CST FALI = (FA_damaged_ − FA_non-damaged_) / (FA_damaged_ + FA_non-damaged_). ^†^Corrected FA values using non-damaged CST FA.

## Discussion

This study demonstrated that CC structural integrity provides complementary information beyond CST integrity in explaining upper limb motor outcome in moderate-to-severe stroke. In hierarchical regression models, CC-FA ~ corrected~ of the genu showed a consistent nominal incremental contribution beyond CST FALI, age, and lesion volume at both 3 and 6 months post-stroke, whereas the body and splenium did not reach statistical significance after accounting for these covariates. As these findings are based on nominal *p* values derived from multiple related models, they require confirmation in larger prospective cohorts. In exploratory analysis, CC-FA ~ corrected~ of the genu was the only variable significantly associated with ΔSAFE, whereas the CST FALI was not. These findings support a network-level perspective in which interhemispheric white matter pathways complement the CST in explaining variability in post-stroke motor outcome.

### The role of CC in interhemispheric connectivity

Patients with more preserved interhemispheric structural connections, reflected by higher CC integrity, exhibited better upper limb motor outcomes with the genu demonstrating the strongest independent association in the hierarchical regression models. Prior neurophysiological and functional imaging studies have suggested that interhemispheric processes contribute to motor performance after stroke (7, 22). In this context, preserved callosal microstructure may represent an anatomical substrate supporting distributed interactions between hemispheres, particularly when ipsilesional structural reserve is limited.

Current neuromodulation strategies for patients with low ipsilesional structural reserve often aim to enhance contralesional excitability to facilitate compensatory processes (23, 24). However, such interventions may yield unsatisfactory results when CC integrity is reduced, limiting the substrate available for interhemispheric interactions (25). The association observed in this study may reflect the role of callosal pathways in supporting these interhemispheric interactions. However, FA represents a structural index of white matter microstructure rather than a direct measure of interhemispheric signaling, and future studies integrating diffusion-based and neurophysiological measures are warranted.

### Region-specific contribution: the genu

Among the CC subregions, the genu was the only subregion to provide a statistically significant independent contribution to upper limb motor outcome after accounting for CST integrity, age, and lesion volume. Patients with higher structural integrity of the genu demonstrated superior upper limb outcomes, consistent with previous findings suggesting that the anterior portion of the CC is associated with motor outcome (5, 26). As the genu primarily contains fibers interconnecting the bilateral prefrontal cortices (19), these findings imply that the prefrontal cortices (PFCs) play a crucial role in motor recovery beyond their well-known contributions to cognitive functions.

One plausible explanation is that the PFCs may serve as the command center of the motor domain. The PFCs have dense interconnections with the premotor cortices, recognized as secondary motor areas (27). These premotor regions receive afferent input from PFCs, relay signals to the primary motor cortex, and send direct projections to the CST (28, 29). Previous studies have shown that PFC activation increases during paretic hand movements, particularly in patients with more severe impairment (9, 30). Moreover, motor imagery has been shown to activate the PFCs and enhance their excitatory connectivity with the premotor cortices (31). These findings collectively support the role of the PFCs in higher-order movement planning and compensatory motor control. Another explanation may involve the PFCs’ role in motor learning. The PFC is integral to the human motor learning circuit, and stroke patients appear to rely more heavily on these regions (32, 33). Acquiring new motor skills requires the executive functions of the PFC, such as attention and action selection, while retaining those skills involves working memory and visuomotor integration (32, 33). Thus, the PFCs may facilitate motor recovery after stroke by supporting the re-learning of lost motor functions and enabling compensation for residual deficits. These findings carry potential clinical relevance. Early DTI assessment of both CST and CC integrity may provide preliminary structural information relevant to rehabilitation planning in patients with moderate-to-severe stroke, though prospective validation in larger cohorts is needed before clinical application. In particular, patients with preserved genu integrity despite severe CST damage may represent a subgroup with relatively greater capacity for motor improvement, and prospective studies are warranted to examine whether such patients respond differentially to interhemispheric or prefrontal-targeting interventions.

In addition, our exploratory analysis suggested that callosal microstructure may also be related to the magnitude of motor improvement over time. Specifically, CC-FA ~ corrected~ of the genu showed a significant association with ΔSAFE (R = 0.396, *p* = 0.041), whereas the CST FALI, the strongest predictor of motor outcome at both 3 and 6 months, was not significantly associated with ΔSAFE (*p* = 0.097). This exploratory finding raises the possibility that genu integrity is related to subsequent motor improvement, and that interhemispheric structural connectivity may be relevant not only to motor status at a given timepoint but also to the trajectory of motor improvement following stroke. However, this association was derived from an exploratory univariable analysis with a modest effect size in a small sample, and ΔSAFE itself may be influenced by baseline SAFE score as well as floor or ceiling effects; therefore, these findings should be interpreted cautiously. Prospective studies with larger cohorts, standardized imaging timepoints, and repeated functional assessments are needed to clarify whether early callosal integrity independently contributes to the trajectory of motor recovery after moderate-to-severe stroke.

### Region-specific contribution: the body and the splenium

Although CC-FA ~ corrected~ of the body and splenium did not provide statistically significant incremental contributions in the hierarchical models, both subregions showed significant associations with upper limb motor outcomes in simple linear regression. These findings are consistent with prior studies reporting contributions of the body and splenium to post-stroke motor outcome (11, 12). The body mainly contains fibers connecting motor-related regions, including premotor cortices, supplementary motor areas, and primary motor cortices. The association observed in simple regression may therefore reflect shared variance with CST integrity and other covariates, rather than an independent contribution. In contrast, the splenium, comprising fibers interconnecting the parietal, temporal, and occipital cortices, is less directly related to primary motor output. The observed association between splenial integrity and motor outcomes may be attributed to its sensory and cognitive projections. Impaired transmission of visuospatial or cognitive information through the splenium could hinder patients from achieving their maximal motor performance.

### Impact of the overall white matter health on motor outcome

In this study, the integrity of all subregions of the CC was positively associated with upper limb motor outcome at both 3 and 6 months post-stroke in simple linear regression, although only the genu retained independent significance in the hierarchical models. Several studies have reported similar associations between multiple CC regions and motor impairment; however, most were cross-sectional and focused on chronic stroke populations (5, 9). Our study newly demonstrated this relationship in patients within the early subacute phase of recovery.

One possible explanation is that CC integrity may reflect the overall condition of cerebral white matter. Interindividual variability in global white matter health could influence the capacity of distributed cortical networks to support motor performance after stroke. Aging reduces the structural integrity of major tracts, with FA decreases shown to be more pronounced in the CC than in projection tracts (22, 34–36), such that corrected CC FA may partially reflect overall white matter health. Future studies using age-adjusted normative FA data could further clarify these baseline differences. Beyond the CST and CC, alternative pathways including premotor-motor connections and cerebrocerebellar tracts have been implicated in post-stroke motor performance (37–39). Incorporating these pathways into multimodal structural models may further enhance our understanding of motor outcome variability.

### Strengths and limitations

A key strength of this study is that neuroimaging data were obtained during the early subacute phase (within 7 days to 3 months from stroke onset). Most previous studies examining CC integrity and post-stroke upper limb function involved chronic patients over 6 months post-stroke, likely after the major phase of recovery had already occurred. Our findings therefore provide insight into structural factors associated with motor outcome during the active recovery period.

Several limitations of our study need to be considered. First, this was a retrospective study, and the regression models were intended to examine associations rather than to establish validated prognostic models. Second, although SAFE score is a strong predictor of upper limb dexterity (3, 40), it does not capture task-specific functional abilities such as those assessed by the ARAT or WMFT. Additionally, while SAFE is ideally measured within 72 h of stroke onset in the PREP algorithm, our assessments were obtained at approximately 7–14 days post-stroke, later than the recommended timeframe. Third, our study did not include the motor-evoked potentials (MEPs) obtained via transcranial magnetic stimulation. As MEPs are neurophysiologic measures that provide functional insight into interhemispheric balance, future studies incorporating both neurophysiologic and imaging biomarkers may help clarify their interchangeability and refine the understanding of how CC integrity relates to interhemispheric communication. Fourth, the relatively small sample size (*N* = 38) and the broad time window of DTI acquisition (7 days to 3 months post-stroke) may have introduced variability in microstructural measurements and limited the number of covariates that could be included in the regression models. Although variables with established associations with post-stroke motor outcome (age, lesion volume, and CST FALI) were prespecified and forced into the first step of the hierarchical model, additional potentially relevant covariates such as baseline SAFE score, sex, education, lesion side, and time from stroke onset to DTI acquisition could not be simultaneously included without an unacceptable predictor-to-subject ratio. To partially address this limitation, a sensitivity analysis was conducted using a parsimonious hierarchical model in which baseline SAFE score and CST FALI were entered as forced covariates in the first step, with genu CC-FA ~ corrected~ added in the second step. The incremental contribution of genu CC-FA ~ corrected~ did not reach statistical significance at either timepoint (3 months: ΔR^2^ = 0.009, *β* = 0.099, 95% CI [−0.769, 6.358], *p* = 0.120; 6 months: ΔR^2^ = 0.018, *β* = 0.142, 95% CI [−0.917, 9.162], *p* = 0.106), suggesting that the association between genu integrity and motor outcome may partly overlap with early motor severity and warrants further clarification in larger prospective studies. Beyond covariate adjustment, formal lesion masking was not applied to the CC ROIs in the JHU atlas-based analysis; although direct lesion overlap with the CC would be expected to be minimal given its anatomical distance from the typical MCA infarct territory, this remains a methodological limitation. The construction of multiple hierarchical models also introduces the potential for type I error inflation, which was addressed by prespecifying the analytic hierarchy and requiring consistent significance across both timepoints as the primary interpretive criterion. Larger prospective studies with more standardized imaging timepoints, formal lesion masking procedures, and sufficient sample sizes to accommodate comprehensive covariate adjustment are needed to validate and extend these findings.

## Conclusion

Upper limb motor outcome after moderate-to-severe stroke depends on the integrity of multiple neural pathways in addition to the CST. This study demonstrated that CC-FA ~ corrected~ of the genu showed a consistent nominal contribution to upper limb motor outcome beyond CST integrity, age, and lesion volume, providing complementary structural information alongside CST integrity, with an exploratory association also observed for the magnitude of subsequent motor improvement. Preserved genu integrity measured during the early subacute phase was associated with better motor performance at 3 and 6 months post-stroke, highlighting the relevance of callosal microstructure, particularly anterior interhemispheric pathways, in explaining variability in motor outcome when corticospinal damage is substantial.

Early assessment of CC and CST integrity may therefore contribute to a more comprehensive structural framework for interpreting motor outcomes in patients with moderate-to-severe stroke. Combined evaluation of these pathways at the time of rehabilitation admission may provide preliminary information regarding individual structural profiles, which warrants further prospective validation before informing individualized goal-setting and treatment planning. Future studies integrating neurophysiological and diffusion-based biomarkers are warranted to further clarify the relationship between structural connectivity and motor outcome and to inform the development of more integrative models of post-stroke motor function.

## Data Availability

The raw data supporting the conclusions of this article will be made available by the authors, without undue reservation.

## Footnotes

1
http://www.fmrib.ox.ac.uk/fsl
