# Supplementary material for: Association between corpus callosum microstructure and upper limb motor outcome in moderate-to-severe ischemic stroke patients at 3–6 months post-stroke
Source: Front Neurol. 2026 Aug 18;17:1852619. doi: 10.3389/fneur.2026.1852619 (PMC13529446; doi:10.3389/fneur.2026.1852619)

Supplementary Material

Supplementary Figure 1. Scatter plots illustrating the association between the change in Shoulder Abduction and Finger Extension (SAFE) score from baseline to 6 months (ΔSAFE) and corrected fractional anisotropy (FA) of corpus callosum subregions: (A) genu, (B) body, and (C) splenium.

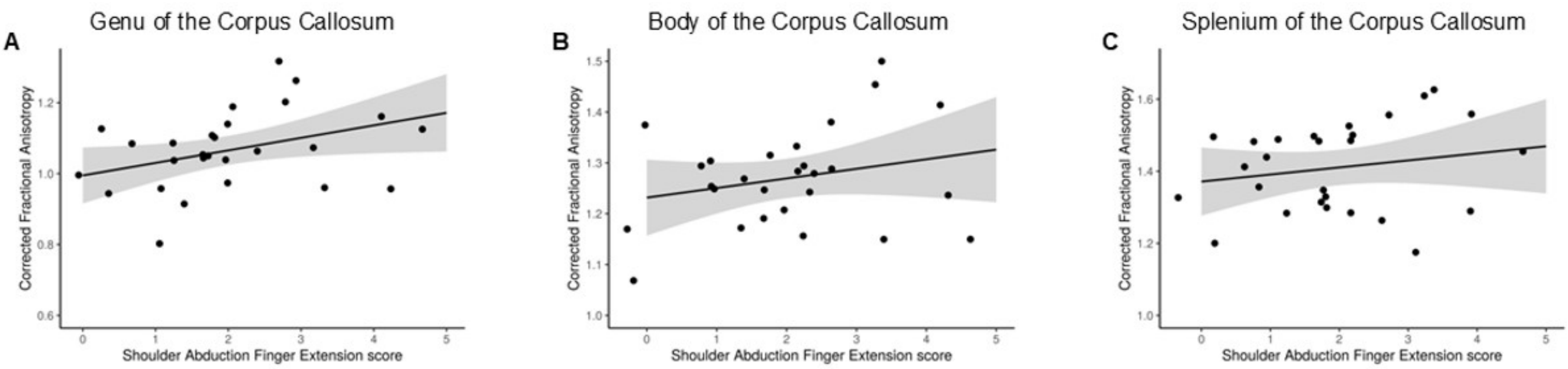

Supplement: Supplementary file 1 [file Image_1.PDF]
